# Knowledge Assessment on the Management of Acute Cor Pulmonale: An Interdisciplinary Survey Study

**DOI:** 10.3390/jcm15072527

**Published:** 2026-03-26

**Authors:** Levin Bolt, Alain Rudiger, Alexander Turk, Mattia Arrigo, Lars C. Huber

**Affiliations:** 1 Department of Internal Medicine, Stadtspital Zurich Triemli, 8063 Zurich, Switzerland; 2 Department of Internal Medicine, Spital Limmattal, 8952 Schlieren, Switzerland; alain.rudiger@spital-limmattal.ch; 3 Department of Internal Medicine, See-Spital Horgen, 8810 Horgen, Switzerland; alexander.turk@see-spital.ch; 4 Division of Internal Medicine, EOC—Ospedale Regionale di Lugano, 6900 Lugano, Switzerland; mattia.arrigo@usi.ch; 5 Facoltà di Scienze Biomediche, Università della Svizzera Italiana, 6900 Lugano, Switzerland; 6 Department of Internal Medicine, University Hospital Zurich, 8091 Zurich, Switzerland; lars.huber@usz.ch; 7 Faculty of Medicine, University of Zurich, 8032 Zurich, Switzerland

**Keywords:** acute cor pulmonale, acute right heart failure, interdisciplinary management, cardiology, emergency medicine, internal medicine, clinical consensus

## Abstract

**Background/Objectives**: Acute cor pulmonale is a critical clinical condition often encountered in acute care settings. Optimal management demands coordinated, interdisciplinary care. The aim of this study was to assess the current knowledge and management strategies for acute *cor pulmonale* among different groups of physicians involved in acute care in Switzerland. **Methods**: A structured questionnaire, extrapolated from the Acute Cardiovascular Care Association of the European Society of Cardiology clinical consensus statement on the diagnosis and treatment of *cor pulmonale*, was distributed among physicians of four specialties. **Results:** A total of 110 physicians participated in this multicenter survey, including 15 “experts,” 71 “generalists” (internal and emergency medicine), and 24 “specialists” (cardiology and intensive care). Experts validated 29 out of 40 questionnaire items (Fleiss Kappa 0.63), which were then used for analysis. Overall, there was substantial agreement with the experts’ answers among non-experts, with most correct response rates exceeding 60%. Significant differences were observed for only two items: experts more frequently recognized the prognostic value of clinical models (87% vs. 59%, *p* = 0.046) and the correct indications for systemic thrombolysis (100% vs. 76%, *p* = 0.037). Between generalists and specialists, differences in knowledge were minimal. Specialists more accurately identified the role of repeated arterial blood gas analysis, while generalists showed better awareness of clinical prognostic models. **Conclusions**: The study highlights a sound knowledge of acute *cor pulmonale* among acute care physicians, regardless of specialty. Despite comparable levels of knowledge, some variations reflect their clinical roles and information sources. The results emphasize the value of existing educational efforts and support the need for comprehensive, accessible guidelines to standardize care in complex conditions like acute *cor pulmonale*.

## 1. Introduction

Acute right ventricular failure caused by a sudden increase in right ventricular afterload, also known as acute cor pulmonale, is a life-threatening condition that can occur in various clinical scenarios. It typically develops in patients with acute pulmonary disorders such as pulmonary embolism, pneumonia, or acute respiratory distress syndrome (ARDS) [1]. The management of patients with acute cor pulmonale involves multiple medical specialties, including emergency medicine, internal medicine, cardiology, and intensive care.

A sound knowledge of the anatomy of the right ventricle (RV) and a thorough understanding of its physiology are essential for making a timely diagnosis and developing individualized treatment strategies [2]. In recent years, increasing attention has been given to RV failure, revealing substantial gaps in knowledge and opportunities for further research, particularly in areas such as pathophysiological mechanisms, hemodynamic monitoring, and clinical methodology [3,4]. Studies have also identified significant misconceptions among clinicians regarding basic principles of RV dysfunction, which can adversely affect patient care [5].

While well-established guidelines from international societies such as the European Society of Cardiology (ESC), the American Heart Association (AHA), and the American College of Cardiology (ACC) focus mainly on left-sided ventricular dysfunction, formal guidelines specifically addressing acute RV failure are missing [6,7]. A recently published clinical consensus statement of the Association for Acute CardioVascular Care of the European Society of Cardiology (ACVC-ESC) provides a framework for the management of patients with acute *cor pulmonale*, including risk stratification, diagnostic approach, and specific treatment [8].

The extent to which this knowledge and management strategies have been implemented into everyday clinical practice remains unclear. The aim of this study was therefore to assess the level of knowledge of the most recent scientific documents among physicians involved in the care of patients with acute *cor pulmonale* and explore whether differences between emergency and internal medicine physicians (“generalists”) and intensive care medicine and cardiology specialists (“specialists”) exist.

## 2. Materials and Methods

### 2.1. Study Design and Participants

The cross-sectional, multicentric survey was conducted in three centers in Switzerland. The electronic questionnaire was made available (i) to internal medicine and emergency medicine physicians (residents and consultants) working at a large tertiary teaching hospital or at two regional referring hospitals (group “generalists”) and (ii) to intensive care medicine and cardiology physicians of the tertiary hospital (group “specialists”).

In addition, members of the acute heart failure study group of the ACVC-ESC or experts who contributed to the Clinical Consensus Statement of the ACVC-ESC were invited to serve as the control group for validation of the questionnaire (group “experts”).

### 2.2. Questionnaire Development and Validation

The survey was anonymized and included seven demographic variables and eight knowledge questions (Q1 to Q8) with five items each, creating a set of 40 statements that could be true or false independently of the others (Supplementary Materials).

The statements were derived from the clinical consensus statement of the ACVC-ESC [8] or other scientific documents of the ESC, which were considered the gold standard. The items have been categorized into four groups according to the following topics: (i) anatomy, physiology, and diagnostic work-up; (ii) monitoring; (iii) treatment; (iv) management of pulmonary embolism.

Each statement was tested for content validity. The group “experts” was used as a reference, and items with an item-level content validity index (I-CVI) of at least 80% and Fleiss Kappa > 0.6 were included in subsequent analysis. The I-CVI quantifies the agreement among experts of an individual item. It was calculated by dividing the number of experts who agreed with the statement by the total number of experts evaluating the item. A high I-CVI indicates substantial agreement among experts.

### 2.3. Data Analysis and Statistics

In a first step, the level of knowledge, expressed as the number and percentage of correct answers for each item, was compared between the “non-experts” (i.e., “generalists” and “specialists” together) and the “experts”. In a second step, the level of knowledge of emergency and internal medicine physicians (“generalists”) was compared with that of intensive care medicine and cardiology specialists (“specialists”).

Values are expressed as median (interquartile range) or as number (percentage), as appropriate. Differences between groups were tested with Fisher’s exact test or Chi-square test for categorical variables and with the Mann–Whitney U-test or Kruskal–Wallis H-test for continuous variables, as appropriate. The null hypothesis was rejected with a two-sided *p*-value < 0.05. For pairwise comparisons, we report *p*-values after Bonferroni correction for multiple testing. All analyses were performed with Excel, Version 16 (Microsoft, Redmond, WA, USA) and SPSS Statistics, Version 29.0. (IBM Corp., Armonk, NY, USA).

## 3. Results

### 3.1. Study Participants

A total of 110 physicians participated in the survey. Of these, 15 belonged to the group “experts”. These answers served for the validation of the questionnaire and as a reference. Among the “non-experts”, 71 physicians were “generalists” (i.e., internal medicine and emergency medicine physicians), and 24 were “specialists” (i.e., cardiology and intensive care medicine physicians). The characteristics of the study participants are provided in Table 1. “Generalists” were younger than “specialists” and “experts” (33 [30, 39] vs. 41 [37, 48] and 44 [40, 62] years, *p* < 0.001), were more frequently in training (61% vs. 33% and 0%, *p* < 0.001), and estimated their competence in managing patients with acute cor pulmonale being lower than “specialists” and “experts” (*p* < 0.001). Accordingly, the main source of information for treating patients with acute *cor pulmonale* was different across the groups (*p* < 0.001). While “generalists” prefer the discussion with colleagues or supervisors followed by online resources, “specialists” and “experts” rely more on guidelines and research articles.

### 3.2. Questionnaire Validation

The group “experts” showed a high agreement (I-CVI > 80%) in 29 of the 40 statements. These items were considered valid (Fleiss Kappa 0.63), and the responses of the “experts” to these statements were designated as the correct answer for subsequent analysis.

The other 11 items were excluded from subsequent analysis. Two statements concerned RV physiology (Q1.2, Q1.4), one was related to ventilatory support in acute *cor pulmonale* (Q5.1), and one to RV support (Q6.3). The remaining seven items addressed pulmonary embolism, spanning the domains of diagnosis (Q3.1, Q3.3, Q3.5), risk stratification (Q7.1, Q7.4), and treatment (Q8.2, Q8.4).

### 3.3. Level of Knowledge Compared to the Expert Group

Overall, the participants showed a substantial agreement with the “experts” on the valid items, with high rates of correct answers mostly exceeding 60% (Table 2). Overall, there was a numerically higher proportion of correct answers among the “expert” group with higher adherence to the current statements of the ACVC-ESC, but the differences did not reach statistical significance (Table 2).

### 3.4. Level of Knowledge Comparing Generalists with Specialists

**Figure 1:**
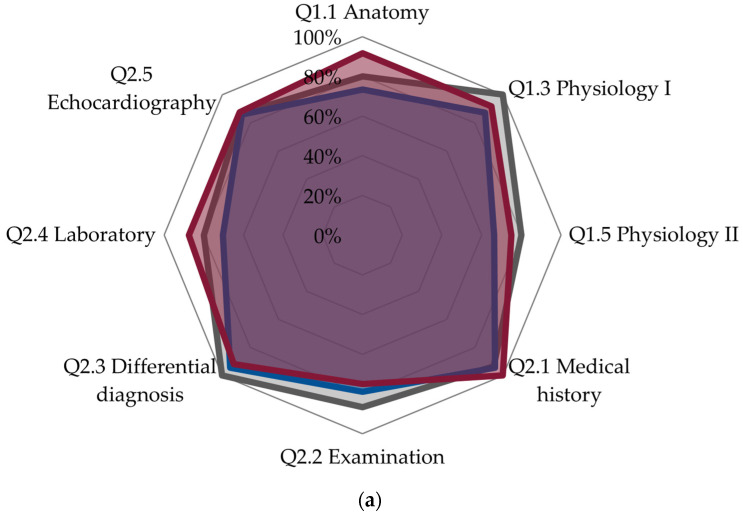

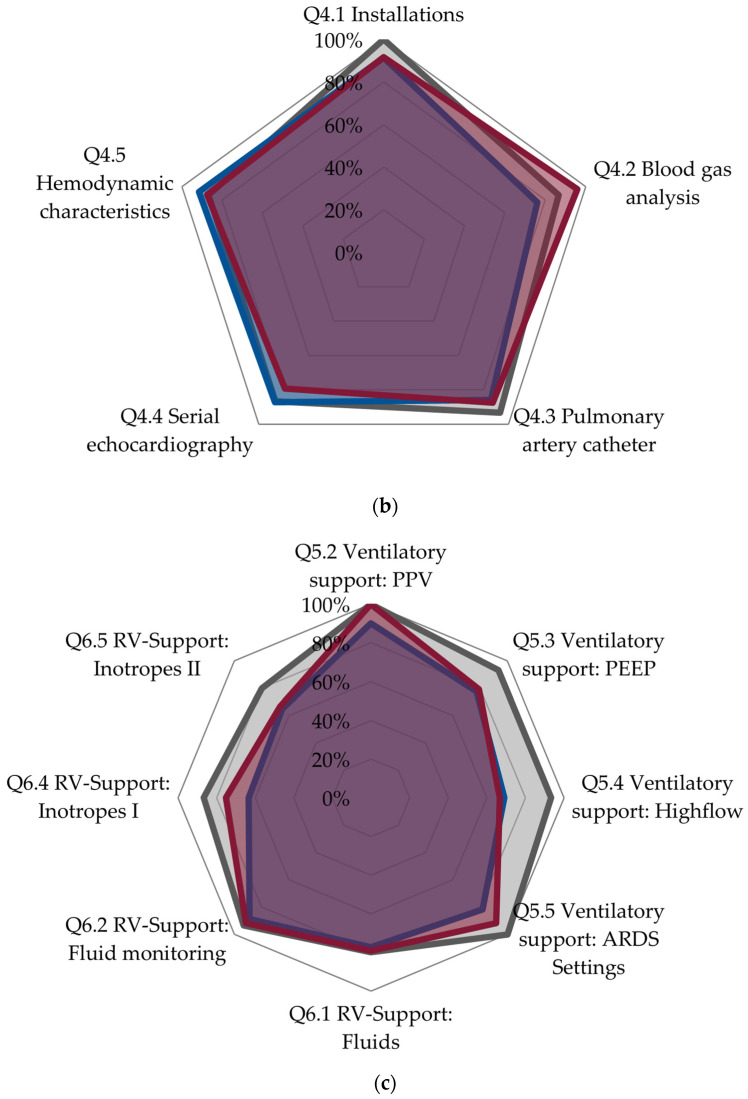

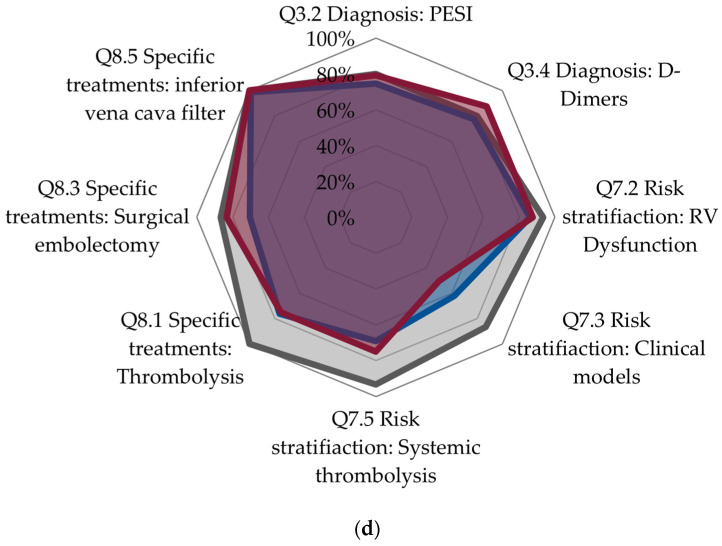
Performance of study participants in the area of (**a**) anatomy, physiology, and diagnostic work-up of acute cor pulmonale; (**b**) monitoring of acute cor pulmonale; (**c**) treatment of acute cor pulmonale; (**d**) diagnosis and management of pulmonary embolism. Legend: gray: experts; blue: generalists; red: specialists.

In general, there were few differences in the level of knowledge between the group of “generalists” and the “specialists” (all *p* > 0.05), Table 2 and Figure 1a–d.

“Specialists” displayed a numerically higher proportion of correct answers on anatomy and physiology of the RV and the role of biomarkers in the diagnosis of acute *cor pulmonale* and pulmonary embolism, but the differences did not reach statistical significance (Table 2, Figure 1a,d). The questions on ventilatory support and inotropic drugs received numerically more correct answers from “specialists”, without reaching statistical significance (Table 2 and Figure 1c). Finally, “specialists” tended to underestimate more frequently the accuracy of clinical models for prognostic purposes in acute pulmonary embolism compared to “generalists” (Table 2 and Figure 1d).

## 4. Discussion

**Figure 2:**
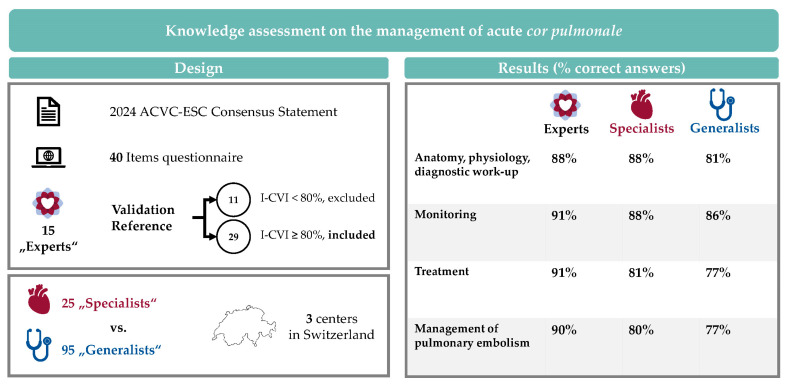
Central illustration.

This multicenter survey provides new insights into the knowledge on the management of acute *cor pulmonale* (illustrated in Figure 2).

First, we observed a substantial agreement overall, both between “experts” and “non-experts”, and between “generalists” and “specialists”. This suggests a common basic understanding of the management of patients with acute *cor pulmonale*, even though “generalists” rated their experience and competence in managing acute *cor pulmonale* lower than “specialists”.

Second, we identified some non-significant knowledge gaps between “generalists” and “specialists”. “Generalists” were more familiar with clinical prognostic models for acute pulmonary embolism, whereas “specialists” demonstrated deeper knowledge of anatomy, pathophysiology, inotropic therapy, and ventilatory support in the context of acute cor pulmonale. These discrepancies likely reflect differences in care settings, clinical exposure, and the primary sources of information used by each group. Generalists are more frequently exposed to the challenges of diagnosis and triage of patients with acute *cor pulmonale*, while “specialists” are more deeply involved in the subsequent management steps. Furthermore, “generalists” primarily relied on peer discussions and online resources, while “specialists” and experts predominantly based their practice on national or international guidelines. To speculate on the reasons for the difference in information sources is beyond the scope of this study. However, “generalists” manage a broad spectrum of conditions under time constraints and may therefore rely more on easily accessible resources and peer consultation for rapid decision-making. Conversely, “specialists” often work in settings where condition-specific, evidence-based protocols are well established. These patterns likely reflect differences in clinical context and workflow rather than divergent attitudes toward evidence-based practice.

Third, all groups demonstrated a generally critical and cautious approach toward the emerging interventional therapies for acute pulmonary embolism, including empirical thrombolysis, insertion of an inferior vena cava filter, and catheter-directed thrombolysis.

The strengths of this study include the expert validation of the questionnaire and participation from both regional and tertiary hospitals. Limitations include its single-country setting and voluntary participation, which may have introduced self-selection bias. Our survey was conducted in Switzerland, so the generalization of the findings may be limited. However, from a methodological perspective, including the dissemination of guideline-based knowledge, we believe that the conclusions are also relevant to other postgraduate healthcare systems. In addition, the questionnaire may not fully capture the complexity of acute *cor pulmonale* and related decision-making. Although participants’ self-assessed levels of expertise were recorded, the analysis did not fully account for differences in clinical experience over the course of the entire career. This bias may have influenced the study results. Of note, 11 of the original 40 items were excluded, likely due to absolute phrasing, lack of consensus (I-CVI < 80% or Fleiss Kappa < 0.6), or variability in clinical experience of the experts.

Previous work by our group has investigated knowledge of the epidemiology, diagnosis, and therapeutic management of patients with heart failure [9]. This study revealed major knowledge gaps and differences between generalists and heart failure specialists. Consequently, the findings of the current study are unexpected. The discrepancies may be due to the fact that only hospital-based physicians were invited to participate in this opinion poll on the management of acute *cor pulmonale*.

Finally, the high proportion of correct answers and the high level of agreement between the different groups of physicians involved in diagnosing and managing patients with acute *cor pulmonale* are positive findings. These results emphasize the importance of creating and implementing guidelines, especially for complex, multidisciplinary medical conditions.

## 5. Conclusions

This study demonstrates substantial knowledge in the management of acute *cor pulmonale* with current educational approaches. These findings highlight the importance of widely accepted and easily accessible guidelines, further supporting standardization and improved patient care. The recently published ACVC-ESC consensus statement is an important step toward this goal; however, further structured efforts are needed to harmonize knowledge, strengthen diagnostic reasoning, and ensure consistent, evidence-based care across diverse clinical settings.

## Figures and Tables

**Table 1:** Characteristics of the study participants.

		Total (N = 110)	Generalists (N = 71)	Specialists (N = 24)	Experts (N = 15)	*p*-Value
Age		35.5 [31, 45]	33 [30, 39]	41 [37, 48]	44 [40, 62]	<0.001
Female sex		51 (46.4%)	38 (53.5%)	8 (33.3%)	5 (33.3%)	0.29
Professional situation	In training	47 (42.7%)	43 (60.6%)	4 (16.7%)	0 (0%)	<0.001
	Staff/consultant	63 (57.3%)	28 (39.4%)	20 (83.3%)	15 (100%)	
How often do you treat patients with acute cor pulmonale?	Less than monthly	60 (54.5%)	43 (60.6%)	12 (50%)	5 (33.3%)	0.054
	Monthly (1–3 cases/month)	38 (34.5%)	19 (26.8%)	12 (50%)	7 (46.7%)	
	Weekly	12 (10.9%)	9 (12.7%)	0 (0%)	3 (20%)	
How do you estimate your competence in managing patients with acute cor pulmonale? (scale from 1 to 5, where 1 indicates the lowest level of competence and 5 indicates highest)	1	8 (7.3%)	8 (11.3%)	0 (0%)	0 (0%)	<0.001
	2	30 (27.3%)	26 (36.6%)	4 (16.7%)	0 (0%)	
	3	39 (35.5%)	26 (36.6%)	10 (41.7%)	3 (20%)	
	4	25 (22.7%)	10 (14.1%)	9 (37.5%)	6 (40%)	
	5	8 (7.3%)	1 (1.4%)	1 (4.2%)	6 (40%)	
Which of the following is your main source of information when treating patients with acute cor pulmonale?	Discussion with colleagues or supervisors	46 (41.8%)	35 (49.3%)	10 (41.7%)	1 (6.7%)	<0.001
	Internal guidelines, SOP, and algorithms of your hospital	8 (7.3%)	7 (9.9%)	0 (0%)	1 (6.7%)	
	Guidelines or similar from national/international societies	31 (28.2%)	9 (12.7%)	12 (50%)	10 (66.7%)	
	Online resources (UpToDate or similar)	22 (20%)	20 (28.2%)	2 (8.3%)	0 (0%)	
	Research articles	3 (2.7%)	0 (0%)	0 (0%)	3 (20%)	

**Table 2:** Performance of study participants in different knowledge areas. The data represent the number and percentage of correct answers for each question.

	Total (N = 110)	Experts (N = 15)	Non-Experts (N = 95)	*p*-Value	Generalists (N = 71)	Specialists (N = 24)	*p*-Value
Acute cor pulmonale: physiology and diagnostic workup							
Q1.1 Anatomy	86 (78.2%)	12 (80.0%)	74 (77.9%)	1.00	52 (73.2%)	22 (91.7%)	0.18
Q1.3 Physiology I	99 (90%)	15 (100.0%)	84 (88.4%)	0.72	62 (87.3%)	22 (91.7%)	1.00
Q1.5 Physiology II	77 (70%)	12 (80.0%)	65 (68.4%)	1.00	47 (66.2%)	18 (75%)	0.92
Q2.1 Medical history	105 (95.5%)	14 (93.3%)	91 (95.8%)	1.00	67 (94.4%)	24 (100%)	1.00
Q2.2 Examination	87 (79.1%)	13 (86.7%)	74 (77.9%)	1.00	56 (78.9%)	18 (75%)	1.00
Q2.3 Differential diagnosis	104 (94.5%)	15 (100.0%)	89 (93.7%)	1.00	67 (94.4%)	22 (91.7%)	1.00
Q2.4 Laboratory	83 (75.5%)	12 (80.0%)	71 (74.7%)	1.00	50 (70.4%)	21 (87.5%)	0.22
Q2.5 Echocardiography	95 (86.4%)	13 (86.7%)	82 (86.3%)	1.00	61 (85.9%)	21 (87.5%)	1.00
Acute cor pulmonale: monitoring							
Q4.1 Installations	102 (92.7%)	15 (100.0%)	87 (91.6%)	1.00	65 (91.5%)	22 (91.7%)	1.00
Q4.2 Blood gas analysis	90 (81.8%)	13 (86.7%)	77 (81.1%)	1.00	54 (76.1%)	23 (95.8%)	0.07
Q4.3 Pulmonary artery catheter	96 (87.3%)	14 (93.3%)	82 (86.3%)	1.00	61 (85.9%)	21 (87.5%)	1.00
Q4.4 Serial echocardiography	94 (85.5%)	13 (86.7%)	81 (85.3%)	1.00	62 (87.3%)	19 (79.2%)	0.66
Q4.5 Hemodynamic characteristics	99 (90%)	13 (86.7%)	86 (90.5%)	1.00	65 (91.5%)	21 (87.5%)	1.00
Acute cor pulmonale: therapy							
Q5.2 Ventilatory support: PPV	103 (93.6%)	15 (100.0%)	88 (92.6%)	1.00	64 (90.1%)	24 (100%)	0.38
Q5.3 Ventilatory support: PEEP	88 (80%)	14 (93.3%)	74 (77.9%)	0.60	55 (77.5%)	19 (79.2%)	1.00
Q5.4 Ventilatory support: high-flow	79 (71.8%)	14 (93.3%)	65 (68.4%)	0.12	49 (69.0%)	16 (66.7%)	1.00
Q5.5 Ventilatory support: ARDS settings	95 (86.4%)	15 (100.0%)	80 (84.2%)	0.44	58 (81.7%)	22 (91.7%)	0.68
Q6.1 RV-Support: fluids	86 (78.2%)	12 (80.0%)	74 (77.9%)	1.00	55 (77.5%)	19 (79.2%)	1.00
Q6.2 RV-Support: fluid monitoring	99 (90%)	14 (93.3%)	85 (89.5%)	1.00	63 (88.7%)	22 (91.7%)	1.00
Q6.4 RV-Support: inotropes I	76 (69.1%)	13 (86.7%)	63 (66.3%)	0.28	45 (63.4%)	18 (75%)	0.6
Q6.5 RV-Support: inotropes II	74 (67.3%)	12 (80.0%)	62 (65.3%)	0.76	46 (64.8%)	16 (66.7%)	1.00
Pulmonary embolism							
Q3.2 Diagnosis: PESI	84 (76.4%)	12 (80.0%)	72 (75.8%)	1.00	53 (74.6%)	19 (79.2%)	1.00
Q3.4 Diagnosis: D-dimers	88 (80%)	12 (80.0%)	76 (80%)	1.00	55 (77.5%)	21 (87.5%)	0.76
Q7.2 Risk stratification: RV dysfunction	96 (87.3%)	14 (93.3%)	82 (86.3%)	1.00	61 (85.9%)	21 (87.5%)	1.00
Q7.3 Risk stratification: Clinical models	69 (62.7%)	13 (86.7%)	56 (58.9%)	0.09	44 (62.0%)	12 (50%)	0.68
Q7.5 Risk stratification: Systemic thrombolysis	81 (73.6%)	14 (93.3%)	67 (70.5%)	0.22	49 (69.0%)	18 (75%)	1.00
Q8.1 Specific treatments: thrombolysis	87 (79.1%)	15 (100.0%)	72 (75.8%)	0.07	54 (76.1%)	18 (75%)	1.00
Q8.3 Specific treatments: surgical embolectomy	83 (75.5%)	13 (86.7%)	70 (73.7%)	0.70	50 (70.4%)	20 (83.3%)	0.58
Q8.5 Specific treatments: inferior vena cava filter	109 (99.1%)	15 (100.0%)	94 (98.9%)	1.00	70 (98.6%)	24 (100%)	1.00

## Data Availability

All data generated or analyzed during this study are included in this published article and its Supplementary Information Files.

## Supplementary Materials

The following supporting information can be downloaded at: https://www.mdpi.com/article/10.3390/jcm15072527/s1. Supplementary S1: questionnaire for the knowledge assessment on the management of acute cor pulmonale.
