# Supplementary material for: Knowledge Assessment on the Management of Acute Cor Pulmonale: An Interdisciplinary Survey Study
Source: J Clin Med. 2026 Mar 26;15(7):2527. doi: 10.3390/jcm15072527 (PMC13073427; doi:10.3390/jcm15072527)
Supplement: Supplementary file 1 [file jcm-15-02527-s001.zip › jcm-4204405-supplementary.pdf]

Supplementary

Supplementary 1: Questionnaire for the knowledge Assessment on the Management of Acute cor pulmonale

Q1. Which of the following sentences about the physiology of the right ventricle (RV) are true?

1. The RV has fewer muscle fibers, is more compliant, and accommodates larger variations in venous return compared to the left ventricle (LV). (C)
2. The RV is more sensitive to changes in afterload compared to the left ventricle (LV).
3. RV myocardial perfusion occurs during diastole only and is less vulnerable to increases in wall tension and systemic hypotension than LV perfusion. (I)
4. The most common cause of acute RV failure is ARDS, followed by acute-on-chronic decompensations and pulmonary embolism.
5. Acute RV dilation with systo-diastolic leftward shift of the septum result in LV compression with reduction in stroke volume and systemic hypotension. (C)

Q2. Which of the following sentences about the diagnostic workup by acute cor pulmonale are true?

1. The history of the patient's present illness combined with signs of RV failure should raise suspicion. (C)
2. Evidence of jugular vein distension reflects volume status rather than RV dysfunction. (I)
3. Differential diagnoses such as pericardial tamponade, acute right ventricular myocardial infarction, and pneumothorax should be ruled out. (C)
4. Elevated high-sensitivity troponin can reveal myocardial injury due to increased RV afterload. (C)
5. The echocardiographic quantification of the RV function can be reliably assessed with one parameter (i.e., tricuspid annular plane systolic excursion, TAPSE). (I)

Q3. Which of the following sentences about the diagnosis of pulmonary embolism (PE) in acute cor pulmonale are true?

1. The pretest probability for the presence of PE estimated by experienced clinicians is non-inferior to the probability calculated by scoring systems.
2. A low PESI score makes the diagnosis of pulmonary embolism unlikely. (I)
3. The prevalence of acute pulmonary embolism is increased in patients presenting with dyspnea when a McQuinn-White sign (SIQIIITIII) is present in ECG.
4. Normal age-adapted levels of D-dimers preclude the use of computed tomography of the chest in patients with acute right ventricular failure. (I)
5. Chest imaging in patients with acute right-sided HF caused by tumor microemboli might yield false negative results.

Q4. Which of the following sentences about the monitoring in acute cor pulmonale are true?

1. Patients with cardiogenic shock due to acute RV failure should receive arterial and central venous lines as standard of care. (C)
2. Arterial blood gas analysis (BGA) should be repeated at regular intervals to assess the effective correction of hypoxaemia, hypercarbia, and acidaemia. (C)
3. The use of pulmonary artery catheter is not recommended. (I)
4. Serial echocardiography determining right- and left-sided filling pressures and cardiac output is not recommended. (I)
5. Elevated central venous pressure (>15 mmHg), discordant right to left-sided filling pressures, low pulmonary artery pulsatility index, and low RV stroke work index support the diagnosis of acute RV failure. (C)

Q5. Which of the following sentences about the ventilatory support in acute cor

pulmonale are true?

1. Hypoxemia, hypercarbia, and acidemia independently produce pulmonary vasoconstriction.
2. Positive pressure ventilation reduces pulmonary vascular resistance (PVR) and intrathoracic pressures, increasing venous return and consequently RV preload. (I)
3. Addition of positive end expiratory pressure (PEEP) reduces the effects of positive pressure ventilation throughout the respiratory cycle. (I)
4. In pulmonary embolism, high-flow nasal cannula and avoidance of positive pressure ventilation where possible are recommended. (C)
5. In ARDS protective ventilation with high tidal volumes, high PEEP and high plateau pressure or driving pressures are recommended. (I)

Q6. Which of the following sentences about RV support in acute RV failure are true?

1. In the majority of patients with acute RV failure, fluid administration is beneficial. (I)
2. Volume administration should be guided by echocardiography, central venous or pulmonary arterial pressure monitoring, and/or cardiac output measurements. (C)
3. Noradrenaline improves systemic hemodynamics and coronary perfusion with minimal effect on pulmonary vascular resistance.
4. In case of hypotension inotropes or inodilators are recommended as first-line therapy. (I)
5. The use of inotropes should ideally be confirmed by measures of inadequate cardiac output despite restoration of blood pressure. (C)

Q7. Which of the following sentences about risk stratification in pulmonary embolism are true?

1. More than one third of patients with acute pulmonary embolism have acute RV dysfunction.
2. Acute RV dysfunction in the context of acute pulmonary embolism confers a four- to fivefold increased risk of death. (C)
3. Clinical models (e.g. PESI score) have excellent diagnostic properties for the identification of patients with pulmonary embolism at low risk of death. (C)
4. The routine use of biomarkers and/or echocardiographic assessment in all patients with acute pulmonary embolism has been shown to be cost-effective and beneficial in reducing mortality.
5. The identification of intermediate-high risk pulmonary embolism is crucial because there is strong evidence supporting systemic thrombolysis in the patient group. (I)

Q8. Which of the following sentences about specific treatments in pulmonary embolism are true?

1. In addition to anticoagulation and supplementary oxygen, systemic thrombolysis is reserved for patients presenting with high-risk PE (i.e., shock). (C)
2. Empirical thrombolysis should be applied in all patients with out-of-hospital cardiac arrest with suspected pulmonary embolism.
3. Surgical embolectomy is recommended for patients with high-risk PE in whom thrombolysis is contraindicated or has failed. (C)
4. Catheter-directed thrombolysis and/or thrombus aspiration (e.g., Flowtriever) have both shown to reduce mortality compared to anticoagulation alone in intermediate-high risk pulmonary embolism.
5. The routine use of inferior vena cava filter is preferred over anticoagulation in case of recurrent PE and should be kept in place for at least 3 months. (I)

Legend: Items in orange showed an item-level content validity index of less than 80% in the group “experts” and were excluded in the subsequent analysis. The answers to the included statements are marked with “(C)” for correct or “(I)” for incorrect accordingly.
